# The influence of proline on surface interactions in aqueous solutions

**DOI:** 10.1016/j.bpj.2025.09.043

**Published:** 2025-10-01

**Authors:** Kieran J. Agg, James E. Hallett, Susan Perkin

**Affiliations:** 1 Physical and Theoretical Chemistry Laboratory, Department of Chemistry, University of Oxford, Oxford, UK; 2 Department of Chemistry, School of Chemistry, Food and Pharmacy, University of Reading, Reading, UK

## Abstract

The amino acid proline is accumulated in a variety of plant species in response to environmental stresses, such as high salinity and extreme temperatures. Although the colligative role of proline as an osmoprotectant is well known, its influence on molecular interactions within the cell has received less attention. Here, we investigate the effects of proline on interaction free energies in aqueous environments, and we find that the presence of proline significantly enhances the repulsive force between charged surfaces relative to pure water. At elevated concentrations, proline alters the short-range, structural interaction, forming layers at the surfaces. In the presence of proline and salt, the near-surface hydration structure is disrupted compared to salt solutions without proline. Overall, we observe that the far-field component of the interaction is relatively insensitive to proline concentration above a low threshold, and the results show that proline contributes to maintaining repulsive colloidal interactions while allowing for tuning of osmotic pressure over a wide spectrum of osmolarity.

## Significance

In addition to its role as a building block for proteins, the amino acid proline is frequently accumulated as individual molecules in the internal fluid of plant cells, particularly those that have evolved to survive in challenging environments, such as high salinity. The role of proline in balancing the external osmotic pressure of the surrounding environment is well known. However, our results show that proline can act to maintain the electrostatic repulsion between charged surfaces in biological systems across a range of concentrations, as well as tuning the interactions at small separations.

## Introduction

Proline, a proteinogenic amino acid, plays a significant role as an osmoprotectant in a variety of plant species (1,2,3). In particular, organisms exposed to environmental stresses such as high salinity, drought, extreme temperatures, and UV radiation have been found with elevated proline levels (4,5,6,7). In one example among vascular halophytes, free proline was found to be present in quantities of up to 10%–20% of the dry shoot weight of *Triglochin maritima* (8).

As for other osmolytes, also called compatible solutes, proline is known to protect these organisms living in salt-stressed environments, primarily by matching the osmotic pressure of the cellular fluid with that of the external growth environment, thus providing a barrier against dehydration (9,10). It has also been shown to have additional protective properties, including by acting as a free radical scavenger, therefore providing protection against oxidative stress (11). Inspired by its utilization in biological organisms, it has even been shown to be beneficial in agricultural contexts: proline provided exogenously to plants in salt-stressed environments has been found to enhance the growth rate of crops (12).

It is essential for cellular function that the accumulation of proline in the cytosol in response to external osmotic stress should not be deleterious to cellular activity. Indeed, enzyme activity was found to be relatively insensitive to wide changes in proline concentration for a range of halophyte enzymes (8). However, questions remain as to whether, and how, osmolytes such as proline modulate interactions within the dense cellular environment. As an electrically neutral zwitterion, proline might be expected to simply contribute toward the “dielectric background” and not significantly modify inter-particle interactions. However, recent works studying more complex osmolytic systems have revealed a strong influence on particle-particle interactions (13,14,15). In another recent example, proline was observed to directly modulate the strength of protein-protein interactions, rendering the interactions more repulsive even at millimolar concentrations (16,17). Furthermore, proline has been shown to control the formation of liquid-liquid-phase-separated droplets (biomolecular condensates) in vivo (18).

**Figure 1:**
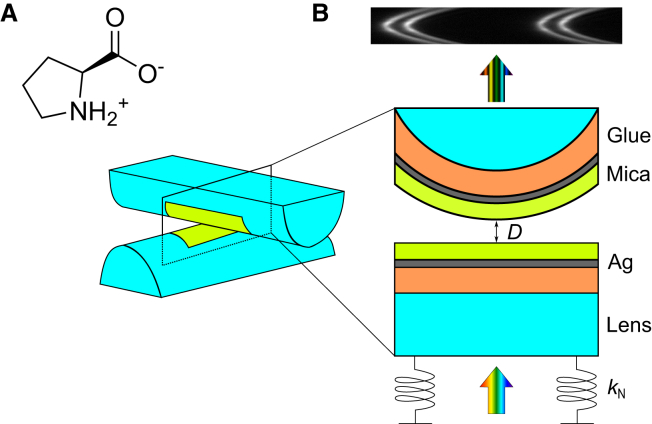
(*A*) The molecular structure of proline. (*B*) Schematic diagram of the surface force balance (SFB) setup showing the light path through the interferometric cavity comprising a crossed-cylinder lens arrangement. The lower lens is mounted on a spring with a known spring constant.

In this work, we report on model experiments directly measuring the interaction between charged surfaces across aqueous solutions containing proline (Pro). We use the surface force balance to investigate whether zwitterionic proline influences surface interactions. We make measurements with and without additional salt to reveal potential synergies or competing effects between the different ionic (KCl) and zwitterionic (proline) solutes present. Measurements of the interaction force as a function of separation distance are made between atomically smooth muscovite mica surfaces that become negatively charged when immersed in the electrolyte solution; these model ultra-smooth surfaces allow us to observe details of the interaction with submolecular-scale (0.1 nm) distance precision. The chemical structure of zwitterionic proline and a schematic of the surface force balance setup, which has previously been described in detail (19), are shown in Fig. 1.

## Materials and methods

In brief, back-silvered mica pieces are glued onto cylindrical glass lenses of a radius *R* ≈ 10 mm. White-light interferometry is used to determine, to subnanometer resolution and 0.1 nm precision, the distance *D* between the surfaces. Bridged by a droplet of the solution of interest, the upper lens is driven at a constant speed toward a lower lens mounted on a spring of constant *k*_N_. Any force *F* acting between the surfaces can thus be determined from the measured deflection of the spring. In the Derjaguin approximation, when *R* ≫ *D*, the interaction free energy between parallel plates can be calculated by the simple relation *W*^‖^(*D*) = *F*(*D*)/2*πR* (20). Further details regarding the experimental setup and methodology are included in Section S1, along with a discussion of errors in Section S2, of the supporting material. The solutions were freshly made before every measurement and used within an hour of preparation. L-proline (Sigma-Aldrich, St. Louis, Missouri, BioUltra, ≥99.5%) and KCl (Thermo Scientific, Waltham, Massachusetts, Puratronic, 99.997%) were used as received. Samples were prepared by weighing the solutes into a flask and mixing with ultrapure water (Milli-Q IQ 7003, 18.2 M Ω.cm, TOC < 3 ppb). In this work, concentrations of the investigated solutions are reported with the unit molal (m), the moles of solute per kilogram of water. The pH values of the measured solutions in this work were all in the range 5.6–6.2. This pH is slightly lower than seven due to the presence of dissolved CO_2_ at atmospheric pressure, producing carbonic acid in small concentrations. Proline has p*K*_a_ values of 1.95 (carboxyl) and 10.64 (amino) and therefore has an isoelectric point of 6.30 (21,22). In other words, in all of our measurements, proline exists in its electrically neutral, zwitterionic form, as displayed in Fig. 1 *A*.

## Results and discussion

**Figure 2:**
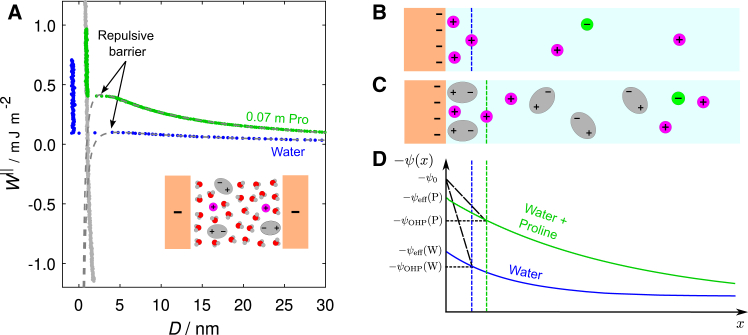
(*A*) Measurement of the interaction free energy *W*^‖^ as a function of surface separation *D* between negatively charged mica surfaces, made across a solution containing 0.07 m proline (*green*) and pure water (*blue*). For the proline solution, the interaction potential measured upon retraction of the surfaces is also shown in gray. The DLVO fits to both measurements are shown by the gray dashed curves. The same data are presented on a log-linear axes in Fig. S1 (Section S3 of the supporting material). (*B* and *C*) Schematics illustrating the structure of a charged interface in pure water and in the presence of proline zwitterions. (*D*) An illustrative plot of potential *ψ* as a function of distance from the interface *x* in both cases. The actual surface potential at the interface *ψ*_0_, the effective potential extrapolated from the electric double-layer potential *ψ*_eff_, and the outer Helmholtz plane (OHP) potential *ψ*_OHP_ are shown; these differ because the decay of potential across the Stern layer is not captured in the Poisson-Boltzmann equation.

The measured interaction free energy as a function of the separation between two negatively charged mica surfaces across an aqueous solution of proline at a low concentration (0.07 m) with no added salt is shown in Fig. 2 *A*. Also shown in this figure is a control experiment with pure water. Upon approach, the surfaces repel one another from large separations, with the repulsion increasing in strength as the separation decreases. This repulsion continues to increase down to a surface separation of around 4 nm, at which point a strong attractive force dominates, and the surfaces jump together. The presence of a strong attraction (energy minimum) is confirmed upon separation of the surfaces, where a negative interaction energy of around −1 mJ m^−2^ is required to bring the surfaces out of contact, at which point there is a sudden jump to large surface separations. Compared to the pure water measurement, the repulsive force is of substantially larger magnitude.

To interpret these measurements, we consider the Derjaguin-Landau-Verwey-Overbeek (DLVO) theory of colloidal stability, which states that the total interaction energy between two surfaces is the sum of electrostatic and van der Waals terms. When identical surfaces are interacting across an electrolyte, the repulsive electrostatic term emerges from the overlap of the double layers of countercharge present at each surface and the resulting excess osmotic pressure. This can be captured using the Poisson-Boltzmann equation and weak overlap approximation, combined with suitable boundary conditions. In the present case, we find that neither a constant charge (CC) nor a constant potential boundary condition properly fits the data; rather, it is necessary to consider charge regulation effects arising from adsorption of ions to the surfaces during the approach and concomitant variation of the surface charge and potential with distance. Here, we employ the constant regulation approximation, which has been used to successfully interrogate a range of measurements across aqueous electrolyte solutions (23,24) and allows for linear regulation between the constant potential and CC boundary conditions with a single dimensionless regulation parameter, *p*, taking values between 0 and 1 (25,26). The value of *p* provides an indication of the molecular processes occurring during approach of the surfaces, as will be illustrated later. The attractive van der Waals contribution to the total interaction free energy is calculated using the Hamaker approach, which is discussed further in Section S4 of the supporting material (27). The resulting total interaction energy can be written as shown in Eq. 1:

(1) $$W^{\|}=-\frac{A}{12\pi D^{2}}+2\epsilon_{0}\epsilon_{e}\kappa_{D}\psi_{\text{eff}}^{2}\frac{e^{-\kappa_{D}D}}{1+\left(1-2p\right)e^{-\kappa_{D}D}}\text{,}$$

where *A* is the Hamaker constant, ϵ_0_ is the permittivity of free space, ϵ_e_ is the electrolyte relative permittivity, *ψ*_eff_ is the effective surface potential, and $\kappa_{D}^{-1}$ is the Debye-Hückel screening length.

Fits to the measurements with pure water and 0.07 m proline in water using Eq. 1 are shown by the gray dashed curves in Fig. 2 *A*. The chosen functional form for the interaction potential provides an accurate fit to the long-range repulsion, as well as providing an attractive force that can explain the jump at small surface separations. The parameters resulting from this and all fits are displayed in Table 1 and reveal a measured Debye-Hückel screening length $\kappa_{D}^{-1}=40\pm 2$ nm.

**Table 1:** Parameters used for DLVO fits to the measured interaction free energy, *W*^‖^, for the displayed measurements across the electrolytes containing proline and potassium chloride

Figure	*c*_Pro_/m	*c*_KCl_/m	*ψ*_eff_/mV	$\kappa_{D}^{-1}$/nm	*p*	$\kappa_{D,\text{pred}}^{-1}$/nm
2	–	–	38 ± 12	65 ± 21	0.88 ± 0.10	–
2 and 3	0.07	–	63 ± 5	40 ± 2	0.90 ± 0.01	–
3	0.35	–	55 ± 2	34 ± 7	0.92 ± 0.02	–
3	0.90	–	49 ± 2	30 ± 5	0.92 ± 0.03	–
4	0.46	0.01	49 ± 3	3.0 ± 0.2	0.61 ± 0.08	3.3
4^a^	–	0.01	29 ± 1	3.8 ± 0.8	1.0 ± 0.0	3.1

For each concentration, the fitted effective surface potential *ψ*_eff_, Debye screening length $\kappa_{D}^{-1}$, and charge regulation parameter *p* are shown. In solutions where KCl is present, the predicted Debye screening length $\kappa_{D,\text{pred}}^{-1}$ is also shown.

a The quality of the fit for this measurement is lower than for the others, and as such, the derived parameters may be less reliable.

Despite there being little difference in the Debye screening length between the proline and pure water cases, there is a significant enhancement to the effective surface potential in the presence of proline: *ψ*_eff_ = 62 ± 5 mV (≈2.5*k*_B_*T*/*e*) for 0.07 m proline and *ψ*_eff_ = 38 ± 14 mV (≈1.5*k*_B_*T*/*e*) for pure water. This strong enhancement of the repulsive force with zwitterions in solution is in line with previous studies, where the enhanced *ψ*_eff_ was attributed to the adsorption of zwitterions at the interface due to the favorable interaction between the negatively charged surface and the strong zwitterion dipole moment (28). The interfacial zwitterions replace the ions that would otherwise be present in the Stern layer, and the cations have a reduced affinity for the plane of negative charge at the edge of the zwitterion layer than for the charged mica interface, thus resulting in a more negative effective potential relative to the pure water measurement. This difference is illustrated schematically in Fig. 2, *B* and *C*, and graphically in Fig. 2 *D*. The enhanced repulsive interaction in the presence of proline relative to pure water is illustrated clearly in Fig. S2 (Section S5 of the supporting material), where both *ψ*_eff_ and the pre-exponential factor $2\epsilon_{0}\epsilon_{e}\kappa_{D}\psi_{\text{eff}}^{2}$ are plotted for these measurements.

The fitted charge regulation parameter is found to be *p* = 0.90 ± 0.03 and indicates that, for 0.07 m proline, the system remains close to the CC boundary during the approach of the surfaces. This is likely a result of the very small concentration of free ions present in the solution (6 × 10^−5^ M, calculated from $\kappa_{D}^{-1}$) available to adsorb during the approach of the surfaces. In light of this, it is meaningful to report the surface charge *σ*_eff_, which can be calculated from the effective surface potential using the Graheme equation, a discussion of which is provided in Section S6 of the supporting material. For this measurement, we calculate the surface charge to be *σ*_eff_ = 7.1 × 10^−3^ e nm^−2^.

**Figure 3:**
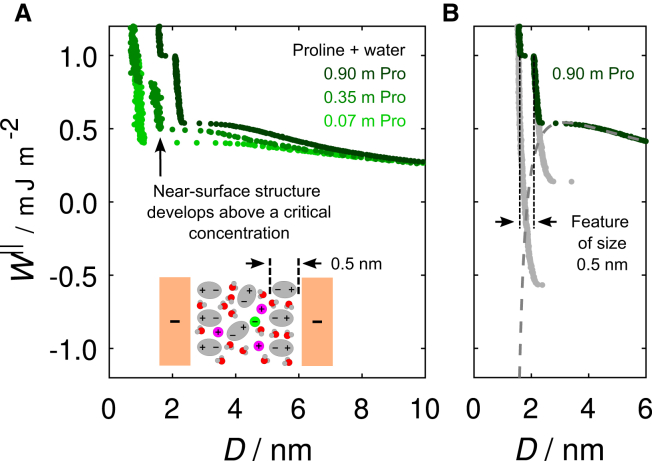
(*A*) Measurement of the interaction free energy *W*^‖^ as a function of surface separation *D* at three different proline concentrations. Additional near-surface features are observed at the higher concentrations (0.35 and 0.90 m), which are not seen at the lower concentration. For clarity, no DLVO fits are shown here, but the associated parameters are displayed in Table 1. (*B*) View of the measurement across the 0.90 m proline solution, concentrated on the near-surface features with the 0.5 nm step indicated. In addition to the approach measurement (*dark green*), the data obtained upon retraction from the two layers are also displayed in gray, which reveal the interaction minimum associated with each layer. The DLVO fit to this measurement is also shown by the gray dashed curve.

Next, we describe experiments at higher proline concentrations. Fig. 3 *A* displays the previously described measurement at a proline concentration of 0.07 m, in addition to measurements performed at elevated concentrations of 0.35 and 0.90 m. At all concentrations, we observe a similar long-range repulsion as the surfaces approach. Interestingly, the longer-ranged force between the surfaces across proline solutions is remarkably insensitive to the proline concentration (above a low threshold; in our experiments, the lowest concentration studied is 0.07 m). Higher proline concentrations give rise to more adsorbed layers and stronger short-range forces, but the far field is not affected, as shown clearly in Fig. S2 (supplemental section 5). Therefore, proline could be used to tune the osmotic pressure (Table S3 in Section S7 of the supporting material displays an estimate for all studied solutions) while retaining close regulation of far-field interaction forces.

At the elevated concentrations (0.35 and 0.90 m), however, we observe an additional feature at small surface separations not present at the lowest concentration, which is displayed more clearly in Fig. 3 *B*. In both cases, a hard wall in the interaction profile is observed at an intermediate distance, followed by a simultaneous jump of the surfaces and squeeze out of a molecular layer of thickness ∼0.5 nm before the closest approach distance is reached. This step size is consistent with the size of a proline molecule along its axis as obtained from x-ray diffraction structural determination (29). We suggest, therefore, that this structural, non-DLVO feature at small surface separations arises from layers of proline adsorbing with their positively charged ammonium group at the negatively charged mica surface and the negatively charged carboxylate group pointing away from the surface. Our interpretation of proline forming layers at the interface is consistent with our earlier analysis behind the enhanced effective surface potential, as well as other recent discoveries finding layering of zwitterions at charged interfaces (15).

**Figure 4:**
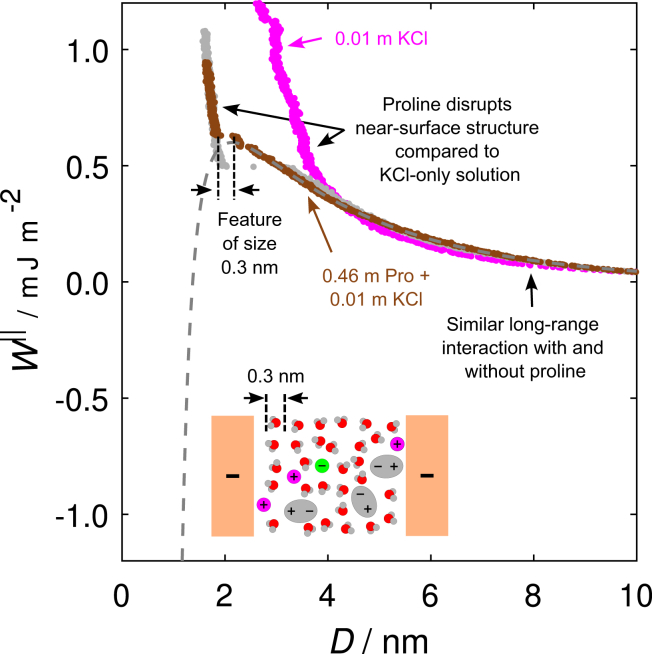
Measurement of the interaction free energy *W*^‖^ as a function of surface separation *D* across a solution containing 0.46 m proline and 0.01 m KCl concentrations (*brown*) and the associated measurement upon retraction of the surfaces (*gray*). The DLVO fit to this measurement is shown by the gray dashed curve. A measurement across a solution containing 0.01 m KCl only (no proline) is displayed in magenta.

Next, we present measurements with added salt across a solution containing 0.46 m proline and 0.01 m KCl in Fig. 4; also displayed for comparison is a measurement across a KCl-only solution at the same salt concentration. From the DLVO fit to the long-range repulsion in the proline-KCl mixture, we see an effective surface potential similar to the proline-only cases (*ψ*_eff_ = 49 ± 5 mV). In contrast, there is a noticeable difference in the Debye screening length ($\kappa_{D}^{-1}=3.0\pm 0.2$ nm) and the charge regulation parameter (*p* = 0.61 ± 0.08). The Debye length can be simply attributed to the presence of KCl, matching that predicted for a 1:1 electrolyte solution at the same KCl concentration (Table 1). Clearly, the reduced value of *p* in the presence of 0.01 m KCl indicates an increased tendency to regulate surface charge during the approach: ions are now more available in solution to adsorb and lower the effective charge. Consistent with this observation, a measurement across 0.33 m proline in the presence of 0.001 m KCl, displayed in Fig. S3 (Section S8 of the supporting material), was found to have an intermediate value between these two cases (*p* = 0.84 ± 0.03).

Turning to the non-DLVO, structural contribution at small surface separations, we see that, in the presence of salt, the short-range “step” characteristic of squeezing out near-surface molecular layers is now reduced to 0.3 ± 0.1 nm. Such features are reminiscent of those present in measurements across dilute aqueous electrolyte solutions (30). These “hydration forces” were measured above a critical salt concentration—4 × 10^−5^ M in the case of KCl (31)—intrinsically linking the origin of these forces to the presence of weakly hydrated ions at the mica interface. Despite proline zwitterions being present at much higher concentrations than the KCl ions, the structural feature is now dominated by the molecular packing of hydrated ions at the interface. Indeed, this observation of a shift in the structural feature from proline dominated to water dominated due to the presence of monovalent ions in the solution is consistent with the appearance of charge regulation behavior across the solutions with salt. Our measurements appear to reveal a link between the observed structural features and the charge regulatory behavior: CC behavior prevails in the electric double-layer repulsive regime when proline dominates the near-surface structure, but charge regulation is enhanced when the water-dominated structural features are observed, owing to the presence of adsorbed ions at the interface.

The measurement across the KCl-only solution shown in Fig. 4 displays a similar long-range interaction to the proline-KCl mixture and similarly displays a repulsion due to hydration forces at short range. However, comparing interactions with and without proline, the magnitude of hydration forces is strongly divergent; in the proline-KCl mixture, the proline zwitterions act to disrupt the near-surface hydration structure. A similar finding has previously been observed in another zwitterionic osmolyte-salt mixture (28). This observation further illustrates the interplay that exists between the proline and ionic species in determining the nature of the overall interaction.

Finally, we return to examine the values of surface potential and surface charge density apparent in each of our experiments, as displayed in Table S2 (Section S6 of the supporting material). It is noticeable that the values of the determined surface potentials vary only slightly with electrolyte composition in the range 1–3 *k*_B_*T*. In contrast, the value of the surface charge in the presence of proline is much more variable and sensitive to ion concentration: values range from *σ*_eff_ ∼ 2 mC m^−2^ with no added KCl to ∼4 and ∼16 mC m^−2^ in the presence of 0.001 and 0.01 m KCl, respectively. This arises from the intrinsic inverse coupling between *σ*_eff_ and $\kappa_{D}^{-1}$ for (relatively) invariant *ψ*_eff_, the latter being set by the ambient thermal energy.

In this work, we have presented a series of experimental measurements across proline-containing aqueous solutions to investigate the role of proline in modulating colloidal interactions. Firstly, our results demonstrate a clear link between proline and an enhanced double-layer repulsion in the absence of salt. Additionally, we find that the short-range interaction, or the non-DLVO structural contribution, is altered by the presence of proline, which forms layers at high concentrations. When salt is present, proline appears to disrupt hydration layers arising from ions at the interface. Finally, with and without salt, the far-field colloidal interaction is stable against varying proline concentration, implying that biomolecular interactions are maintained while osmotic pressure varies over a wide range.

## Data and code availability

All raw data (interaction free energy as a function of distance) are available from the Oxford University Research Archive (https://doi.org/10.5287/ora-00xxnmb7z).

## Acknowledgments

The authors gratefully acknowledge funding from the 10.13039/501100000781European Research Council under grant 101001346 ELECTROLYTE. K.J.A. would like to acknowledge support from the Oxford-The Queen’s College Graduate Scholarship in partnership with the Clarendon Fund, 10.13039/501100000769University of Oxford.

## Author contributions

K.J.A., J.E.H., and S.P. designed the research. K.J.A, and J.E.H. performed the research and analyzed the data. K.J.A., J.E.H., and S.P. wrote the manuscript.

## Declaration of interests

The authors declare no competing interests.

## Supporting material

Document S1. Figures S1–S3, Tables S1–S4, and Sections S1–S8

Document S2. Article plus supporting material
