# Supplementary material for: The influence of proline on surface interactions in aqueous solutions
Source: Biophys J. 2025 Oct 1;124(23):4096–101. doi: 10.1016/j.bpj.2025.09.043 (PMC12709381; doi:10.1016/j.bpj.2025.09.043)
Supplement: Document S1. Figures S1–S3, Tables S1–S4, and Sections S1–S8 [file mmc1.pdf]

Biophysical Journal, Volume *124*

Supplemental information

The influence of proline on surface interactions in aqueous solutions

Kieran J. Agg, James E. Hallett, and Susan Perkin

1 Additional experimental details

In our measurements, the surfaces are initially prepared by manually cleaving mica (optical-grade ruby muscovite mica, S & J Trading Inc.) in a dust free environment, yielding atomically smooth facets of area on the magnitude of several cm^2 and thickness of 2 to 4 $\mu\mathrm{m}$. These are subsequently adhered on a freshly cleaved mica substrate and back-silvered via thermal evaporation to a layer thickness of $\sim$ 45 nm (99.9999%, Alfa Aesar) using an Auto 306 thermal evaporator (HHV Ltd.). The mica substrates are then stored under vacuum until use. Two of these mica pieces are glued using an epoxy resin (EPON™ Resin 1004F, Miller-Stephenson Chemical Co., Inc.), silver-side down, to cylindrical glass lenses of radius $R \approx 10$ mm. The lenses are mounted in a crossed-cylinder orientation, with the lower lens located on a leaf spring of pre-calibrated spring constant. A white light source is incident on an interferometric cavity which is formed by the two silver mirrors, and the light emergent from this cavity is passed into a grating spectrometer to measure the resulting interference pattern which appears as fringes of equal chromatic order (FECO). Analysis of the FECO can determine the surface separation D and the radius of the surfaces R. The forces exerted between the two surfaces can be calculated by measuring the deflection of the lower lens on the leaf spring. A calibration measurement of the mica thickness is performed by bringing the surfaces together in air, which is necessary for the determination of absolute surface separation.

The electrolyte solution of interest is then injected between the two lenses to form a bridging droplet ($\sim$ 0.3 mL). Equilibrium forces were measured by driving the surfaces toward each other at a constant velocity (ranging between 2 and 10 nm s^{-1}) using a mechanical or piezoelectric drive. A measurement performed whilst approaching the surfaces yields data for the monotonic repulsive force at larger surface separations and structural forces at smaller surface separations, and retracting the surfaces can reveal the nature of one or more potential minima.

2 Experimental error analysis

In this work, we choose to present individual surface force measurements for different sample compositions, as these most clearly show features of interest at small length scales. The random error in the measurement of an individual data point within a single run is very small - approximately $\sim$ 0.1 nm in D and $\sim$ 0.01 mJ m^{-2} in $W^{||}$ - thus allowing these molecular-scale features to be visible. These errors arise from the uncertainty in the tracking of the fringes of equal chromatic order (FECO) through which distance is calculated, and in the value of the spring constant from which forces or interaction energies are determined.

If multiple measurement runs were averaged, these subtle features would be smeared out. Systematic errors are present across runs, of magnitude $\sim$ 0.5 nm in D and $\sim$ 0.1 mJ m^{-2}, which arise from errors in optical alignment, measurement of the radius of curvature, temperature and concentration of samples. For the measurements presented in this work for a given composition, no significant differences appear between individual measurement runs, with no dependence on equilibration time (between adjacent runs), time after introducing the liquid between the mica surfaces or the microscopic contact spot between the two crossed cylinders. Between runs, there are small variations in the forces at which jumps due to a spring instability occur and in the magnitude of long range interactions, but changes in distance over which features occur (e.g. molecular layer thicknesses) and screening lengths are more reproducible.

3 Interaction energies presented on log-linear axes

Figs. 2 (A), 3 (A) and 4 from the main text are reproduced in Fig. S1 on log-linear axes. Presenting the data in this manner aids the visualisation of the range and magnitude of the interaction.

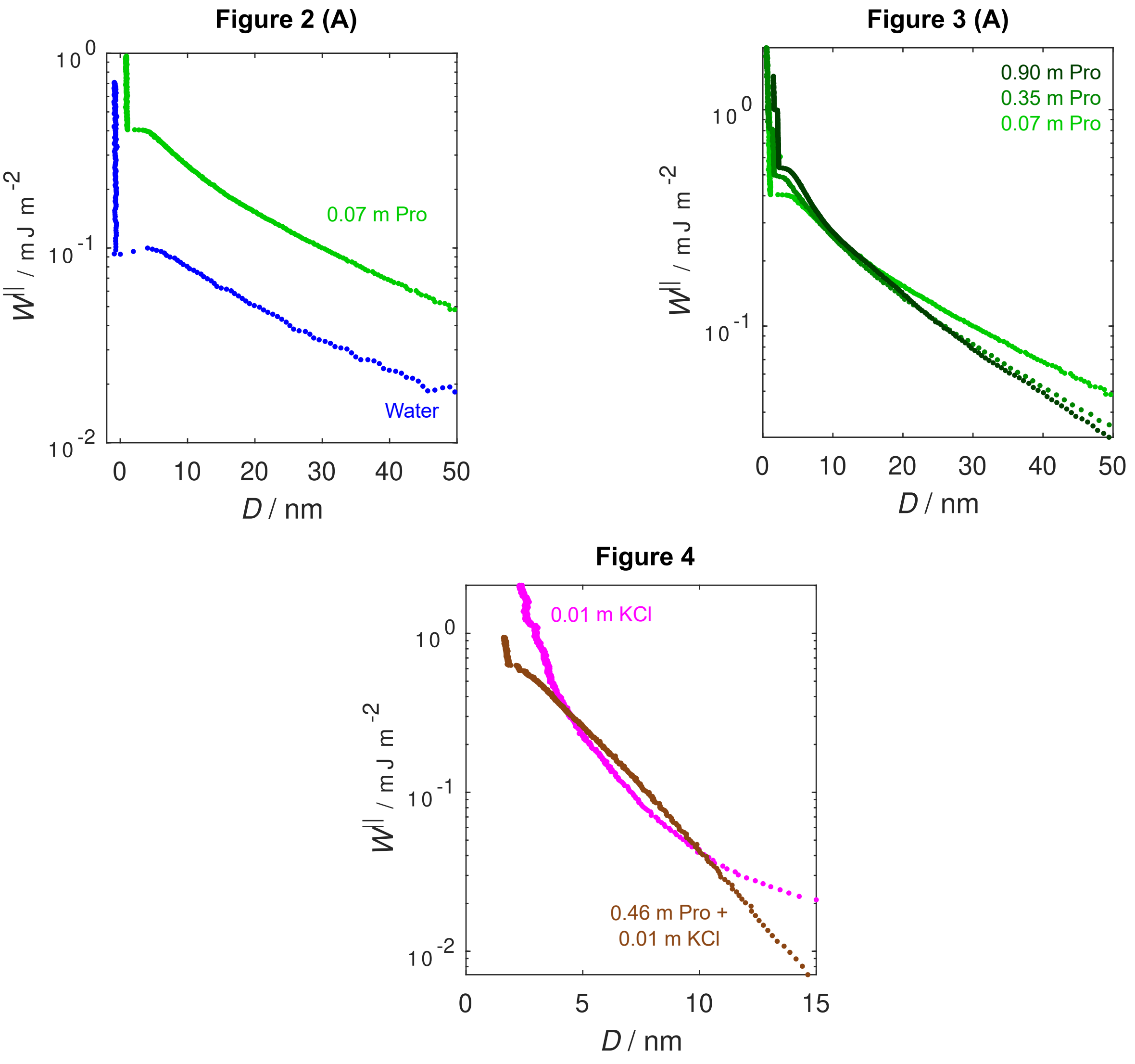

Figure S1. Data from Fig. 2 (A), Fig. 3 (A) and Fig. 4 in the main text are reproduced here on log-linear axes.

4 Calculation of Hamaker constants

For the DLVO fits in this work, we calculate the Hamaker constants between mica surfaces across electrolytes of given composition using the Lifshitz theory with the approach detailed by Israelachvili, as shown in Eq. S1 [1]:

$$A = \frac{3}{4}kT\left(\frac{\epsilon_\mathrm{m}-\epsilon_\mathrm{e}}{\epsilon_\mathrm{m}+\epsilon_\mathrm{e}}\right)^2 + \frac{3h\nu_\mathrm{e}}{16\sqrt{2}}\frac{(n_\mathrm{m}^2-n_\mathrm{e}^2)^2}{(n_\mathrm{m}^2+n_\mathrm{e}^2)^{\frac{3}{2}}} \tag{S1}$$

where k is the Boltzmann constant; T is the temperature (295 K); ϵ_m and ϵ_e are the relative permittivities of mica and electrolyte, respectively; h is the Planck constant; ν_e is the plasma frequency of the free electron gas; and n_m and n_e are the refractive indices of mica and electrolyte, respectively [1,2]. The relative permittivity of mica was taken as 2, the frequency of the free electron gas was taken as 3×10^{15} s^{-1}, and the refractive index of mica was taken as 1.6 [1]. An Abbe 60 Refractometer (Bellingham and Stanley) and a sodium lamp of wavelength 589.3 nm was used to measure the refractive indices of the measured solutions.

The relative permittivity of the electrolyte was estimated using Eq. S2:

$$\epsilon_\mathrm{e} = \epsilon_\mathrm{w} + \delta_\mathrm{Pro}c_\mathrm{Pro} + \delta_\mathrm{KCl}c_\mathrm{KCl} \tag{S2}$$

where ϵ_w is the relative permittivity of water, δ is the dielectric increment or decrement and c is the solute concentration. Here, the dielectric increments or decrements were obtained from literature, and we have taken δ_Pro to be 21.0 / m [3], and δ_KCl to be -8.85 / m. [4]

The calculated Hamaker constants and associated variables are displayed in Table S1.

Table S1. Parameters used for Hamaker constant calculations. Concentrations of proline c_Pro and KCl c_KCl, electrolyte refractive index n_e, electrolyte relative permittivity ϵ_e and Hamaker constant A are shown.

Figure	c_Pro / m	c_KCl / m	n_e	ϵ_e	A / 10^{-20} J
2	-	-	1.3300	78.4	2.11
2 & 3	0.07	-	1.3339	79.9	2.05
3	0.35	-	1.3394	85.5	1.98
3	0.90	-	1.3507	97.3	1.84
S3	0.33	0.001	1.3382	85.3	2.00
4	0.46	0.010	1.3414	88.0	1.96
S3	-	0.001	1.3320	78.4	2.08
4	-	0.010	1.3321	78.3	2.08

5 Surface potential as a function of proline concentration

For the proline-only aqueous solutions discussed in this work (Figs. 2 (A) and 3 (A)), the effective surface potential ψ_{eff} obtained from the DLVO fits, as tabulated in Table 1, is plotted as a function of proline concentration in Fig. S2 (A). Since the magnitude of the repulsive barrier present in the interaction profile is not solely dependent on ψ_{eff}, the pre-exponential factor $2\epsilon_0\epsilon_{\mathrm{e}}\kappa_{\mathrm{D}}\psi_{\mathrm{eff}}^2$ is plotted in Fig. S2 (B). This figure clearly demonstrates the enhanced repulsive barrier in the presence of proline relative to pure water, but that the magnitude is not significantly changed by dialling up the proline concentration.

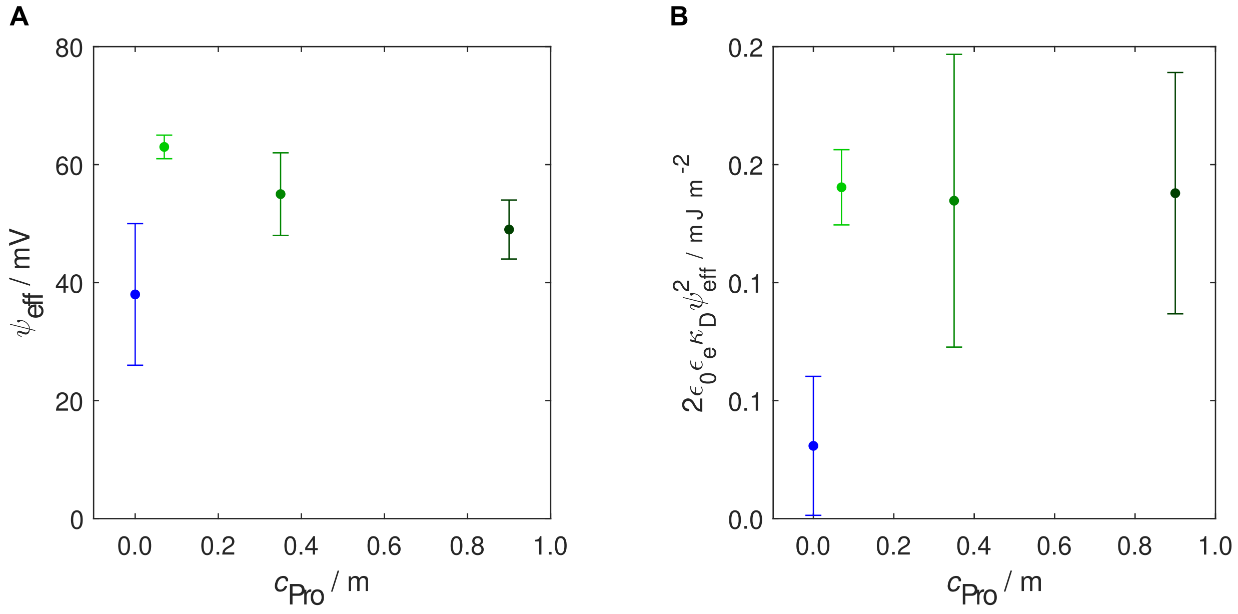

Figure S2. (A) Effective surface potential ψ_{eff} as a function of proline concentration. (B) The pre-exponential factor $2\epsilon_0\epsilon_{\mathrm{e}}\kappa_{\mathrm{D}}\psi_{\mathrm{eff}}^2$ as a function of proline concentration.

6 Calculations of surface charge density

In order to interconvert between the fitted value for effective surface potential ψ_{eff} and surface charge σ, we use the following relations discussed in the review article by Smith *et al.*[5]

For weakly charged surfaces, the effective surface charge density σ_{eff} can be calculated using the Debye-Hückel relation shown in Eq. S3.

$$\sigma_{\text{eff}} = \epsilon_0 \epsilon \kappa_{\text{eff}} \psi_{\text{eff}} \tag{S3}$$

For more highly charged surfaces, the Poisson-Boltzmann equation must be invoked. Eq. S4 relate the effective surface potential ψ_{eff}, extracted from our measurements, and diffuse layer potential ψ_{dl}, the potential at the origin of the diffuse layer, across a symmetric 1:1 electrolyte.

$$\psi_{\text{eff}} = \frac{4kT}{e} \tanh\left(\frac{e\psi_{\text{dl}}}{4kT}\right) \tag{S4}$$

A surface charge density σ can be calculated from the diffuse layer potential ψ_{dl} using the Graheme equation, shown in Eq. S5 for a symmetric 1:1 electrolyte.

$$\sigma = \frac{2kT\epsilon_0\epsilon\kappa_{\text{D}}}{e} \sinh\left(\frac{e\psi_{\text{dl}}}{2kT}\right) \tag{S5}$$

The surface charge densities and surface potentials calculated from the fitted effective surface potential ψ_{eff} and Eqs. S3-S5 are displayed in Table S2.

Table S2. Table of calculated constants for surface potentials and charge densities. Concentrations of proline c_{Pro} and KCl c_{KCl}, fitted effective surface potential ψ_{eff}, effective surface charge density σ_{eff}, double layer potential ψ_{dl} and surface charge density σ are shown. †The quality of the fit for this measurement is lower than for the others, and as such, the derived parameters may be less reliable.

Figure	c_{Pro}/ m	c_{KCl}/ m	κ_{D}^{-1} / nm	ψ_{eff} / mV	σ_{eff} / 10^{-3} e nm^{-2}	ψ_{dl} / mV	σ / 10^{-3} e nm^{-2}
2	-	-	65	38	2.5	40	2.9
2 & 3	0.07	-	40	63	7.1	74	11
3	0.35	-	34	55	7.6	61	11
3	0.90	-	30	49	8.8	54	11
S3	0.33	0.001	11	49	21	53	27
4	0.46	0.010	3.0	49	78	53	100
S3	-	0.001	9.5	56	26	63	36
4 †	-	0.010	3.8	29	33	30	36

7 Osmotic pressures of studied solutions

The osmotic pressure Π can be calculated for an ideal solution using the van't Hoff equation, shown in Eq. S6:

$$\Pi = \sum icRT \tag{S6}$$

which is summed over all solutes, and where i is the van't Hoff factor, c is the solute concentration, R is the ideal gas constant and T is the temperature. The van't Hoff factor i was taken as 1 for proline and 2 for KCl, and the solute concentration in molar was approximated to be the measured value in molal, valid for small concentrations. The osmotic pressure of all solutions studied in this work is shown in Table S3.

Table S3. Osmotic pressures of all solutions studied in this work. Concentrations of proline c_{Pro} and KCl c_{KCl} and osmotic pressure Π are shown.

Figure	c_{Pro} / m	c_{KCl} / m	Π / atm
2	-	-	-
2 & 3	0.07	-	1.7
3	0.35	-	8.6
3	0.90	-	22.0
S3	0.33	0.001	8.1
4	0.46	0.010	11.7
S3	-	0.001	0.05
4	-	0.010	0.49

8 Surface forces measurements at additional concentrations

A measurement across a solution containing 0.33 m proline and 0.001 m KCl is displayed in Fig. S3. The associated DLVO and Hamaker constant parameters are displayed in Table S4 and S1, respectively, along with those from the main article. This measurement appears qualitatively similar to the high concentration proline measurements with a long-range repulsion, van der Waals attractive jump and structural feature in the approach interaction profile. However, the structural jump is of size 0.3 nm, and thus likely dominated by the packing of water molecules at the interface, akin to the structural feature observed in the measurement at the 0.01 m KCl concentration (Fig. 4). The effective surface potential of the long-range attraction is similarly enhanced relative to the pure water case ($\psi_{\mathrm{eff}} = 49 \pm 2$ mV); the Debye screening length is consistent with that for a 1:1 electrolyte containing 0.001 m KCl ($\kappa_{\mathrm{D}}^{-1} = 11 \pm 1$ nm); and the charge regulation parameter is an intermediate value between the proline-only measurements and the 0.01 m KCl measurement, as discussed in the main text ($p = 0.84 \pm 0.03$) .

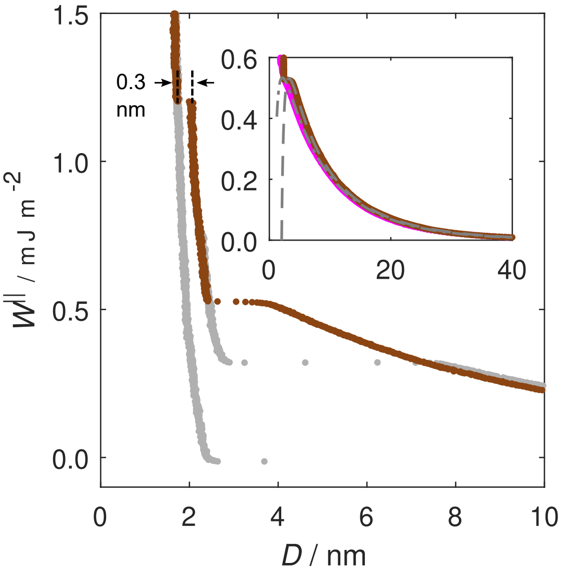

Figure S3. Measurement of the interaction potential ($W^{||}$) across proline solutions as a function of surface separation (D) between negatively charged mica surfaces, made across an electrolyte containing 0.33 m proline and 0.001 m KCl. The measurement of interaction potential measured upon approach of the surfaces is shown in brown, and the retractions from the two layers are shown in grey. In the inset, this proline-KCl measurement (brown) is compared to one across a pure 0.001 m KCl solution (magenta) in addition to the associated DLVO fits (grey).

d

Table S4. Parameters used for DLVO fits to the measured interaction free energy $W^{||}$, for the displayed measurements across the electrolytes containing proline and potassium chloride. For each concentration, the fitted effective surface potential ψ_{eff}, Debye screening length κ_{D}^{-1} and charge regulation parameter p are shown. In solutions where KCl is present, the predicted Debye screening length $\kappa_{\mathrm{D,\ pred}}^{-1}$ is also shown.

c_{Pro}/ m	c_{KCl}/ m	ψ_{eff} / mV	κ_{D}^{-1} / nm	p	D_0 / nm	$\kappa_{\mathrm{D,\ pred}}^{-1}$ / nm
0.33	0.001	49 ± 2	11 ± 1	0.84 ± 0.03	1.4 ± 0.1	10
-	0.001	56 ± 4	9.5 ± 0.9	0.72 ± 0.06	0.2 ± 0.3	9.7

Supporting References

[1] J. N. Israelachvili, Intermolecular and Surface Forces, Academic Press, 3rd edn, 2011.

[2] E. M. Lifshitz, Journal of Experimental and Theoretical Physics, 1956, **2**, 73–83.

[3] T. Chen, G. Hefter and R. Buchner, Journal of Physical Chemistry A, 2003, **107**, 4025–4031.

[4] J. Kirchnerova, P. G. Parrel, J. T. Edward, T. Shida, W. H. Hamill, J. C. Phys, J. L. Franklin, J. G. Dillard, M. Rosenstock, J. T. Herron, K. Draxl, F. H. Field and P. G. Farrell, Journal of Physical Chemistry, 1976, **80**, 1974–1980.

[5] A. M. Smith, M. Borkovec and G. Trefalt, Advances in Colloid and Interface Science, 2020, **275**, 102078.
