# Supplementary material for: The influence of proline on surface interactions in aqueous solutions
Source: Biophys J. 2025 Oct 1;124(23):4096–101. doi: 10.1016/j.bpj.2025.09.043 (PMC12709381; doi:10.1016/j.bpj.2025.09.043)
Supplement: Document S2. Article plus supporting material [file mmc2.pdf]

Biophysical Journal
Article

Biophysical Society

The influence of proline on surface interactions in aqueous solutions

Kieran J. Agg,[1] James E. Hallett,[2] and Susan Perkin[1,*]
[1]Physical and Theoretical Chemistry Laboratory, Department of Chemistry, University of Oxford, Oxford, UK and [2]Department of Chemistry, School of Chemistry, Food and Pharmacy, University of Reading, Reading, UK

ABSTRACT The amino acid proline is accumulated in a variety of plant species in response to environmental stresses, such as high salinity and extreme temperatures. Although the colligative role of proline as an osmoprotectant is well known, its influence on molecular interactions within the cell has received less attention. Here, we investigate the effects of proline on interaction free energies in aqueous environments, and we find that the presence of proline significantly enhances the repulsive force between charged surfaces relative to pure water. At elevated concentrations, proline alters the short-range, structural interaction, forming layers at the surfaces. In the presence of proline and salt, the near-surface hydration structure is disrupted compared to salt solutions without proline. Overall, we observe that the far-field component of the interaction is relatively insensitive to proline concentration above a low threshold, and the results show that proline contributes to maintaining repulsive colloidal interactions while allowing for tuning of osmotic pressure over a wide spectrum of osmolarity.

SIGNIFICANCE In addition to its role as a building block for proteins, the amino acid proline is frequently accumulated as individual molecules in the internal fluid of plant cells, particularly those that have evolved to survive in challenging environments, such as high salinity. The role of proline in balancing the external osmotic pressure of the surrounding environment is well known. However, our results show that proline can act to maintain the electrostatic repulsion between charged surfaces in biological systems across a range of concentrations, as well as tuning the interactions at small separations.

INTRODUCTION

Proline, a proteinogenic amino acid, plays a significant role as an osmoprotectant in a variety of plant species (1–3). In particular, organisms exposed to environmental stresses such as high salinity, drought, extreme temperatures, and UV radiation have been found with elevated proline levels (4–7). In one example among vascular halophytes, free proline was found to be present in quantities of up to 10%–20% of the dry shoot weight of *Triglochin maritima* (8).

As for other osmolytes, also called compatible solutes, proline is known to protect these organisms living in salt-stressed environments, primarily by matching the osmotic pressure of the cellular fluid with that of the external growth environment, thus providing a barrier against dehydration (9,10). It has also been shown to have additional protective properties, including by acting as a free radical scavenger, therefore providing protection against oxidative stress (11). Inspired by its utilization in biological organisms, it has even been shown to be beneficial in agricultural contexts: proline provided exogenously to plants in salt-stressed environments has been found to enhance the growth rate of crops (12).

It is essential for cellular function that the accumulation of proline in the cytosol in response to external osmotic stress should not be deleterious to cellular activity. Indeed, enzyme activity was found to be relatively insensitive to wide changes in proline concentration for a range of halophyte enzymes (8). However, questions remain as to whether, and how, osmolytes such as proline modulate interactions within the dense cellular environment. As an electrically neutral zwitterion, proline might be expected to simply contribute toward the "dielectric background" and not significantly modify inter-particle interactions. However, recent works studying more complex osmolytic systems have revealed a strong influence on particle-particle interactions (13–15). In another recent example, proline was observed to directly modulate the strength of protein-protein

Submitted April 9, 2025, and accepted for publication September 26, 2025.
*Correspondence: susan.perkin@chem.ox.ac.uk
Editor: Ronald Koder.
https://doi.org/10.1016/j.bpj.2025.09.043
© 2025 The Author(s). Published by Elsevier Inc. on behalf of Biophysical Society.
This is an open access article under the CC BY license (http://creativecommons.org/licenses/by/4.0/).

interactions, rendering the interactions more repulsive even at millimolar concentrations (16,17). Furthermore, proline has been shown to control the formation of liquid-liquid-phase-separated droplets (biomolecular condensates) in vivo (18).

In this work, we report on model experiments directly measuring the interaction between charged surfaces across aqueous solutions containing proline (Pro). We use the surface force balance to investigate whether zwitterionic proline influences surface interactions. We make measurements with and without additional salt to reveal potential synergies or competing effects between the different ionic (KCl) and zwitterionic (proline) solutes present. Measurements of the interaction force as a function of separation distance are made between atomically smooth muscovite mica surfaces that become negatively charged when immersed in the electrolyte solution; these model ultra-smooth surfaces allow us to observe details of the interaction with submolecular-scale (0.1 nm) distance precision. The chemical structure of zwitterionic proline and a schematic of the surface force balance setup, which has previously been described in detail (19), are shown in Fig. 1.

MATERIALS AND METHODS

In brief, back-silvered mica pieces are glued onto cylindrical glass lenses of a radius $R \approx 10$ mm. White-light interferometry is used to determine, to subnanometer resolution and 0.1 nm precision, the distance D between the surfaces. Bridged by a droplet of the solution of interest, the upper lens is driven at a constant speed toward a lower lens mounted on a spring of constant k_N. Any force F acting between the surfaces can thus be determined from the measured deflection of the spring. In the Derjaguin approximation, when $R \gg D$, the interaction free energy between parallel plates can be calculated by the simple relation $W^{\|}(D) = F(D)/2\pi R$ (20). Further details regarding the experimental setup and methodology are included in Section S1, along with a discussion of errors in Section S2, of the supporting material. The solutions were freshly made before every measurement and used within an hour of preparation. L-proline (Sigma-Aldrich, St. Louis, Missouri, BioUltra, ≥99.5%) and KCl (Thermo Scientific, Waltham, Massachusetts, Puratronic, 99.997%) were used as received. Samples were prepared by weighing the solutes into a flask and mixing with ultrapure water (Milli-Q IQ 7003, 18.2 M Ω.cm, TOC < 3 ppb). In this work, concentrations of the investigated solutions are reported with the unit molal (m), the moles of solute per kilogram of water. The pH values of the measured solutions in this work were all in the range 5.6–6.2. This pH is slightly lower than seven due to the presence of dissolved CO_2 at atmospheric pressure, producing carbonic acid in small concentrations. Proline has pK_a values of 1.95 (carboxyl) and 10.64 (amino) and therefore has an isoelectric point of 6.30 (21,22). In other words, in all of our measurements, proline exists in its electrically neutral, zwitterionic form, as displayed in Fig. 1 *A*.

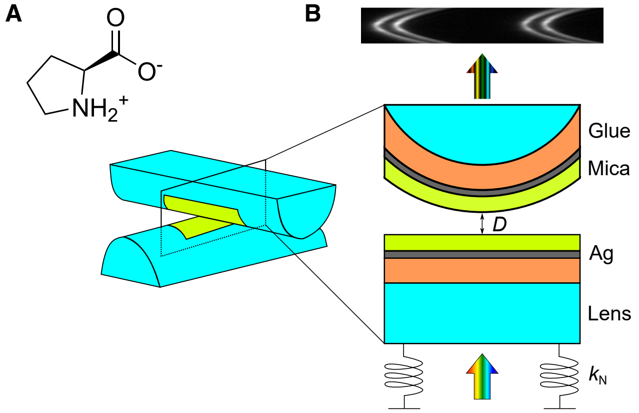

FIGURE 1 (*A*) The molecular structure of proline. (*B*) Schematic diagram of the surface force balance (SFB) setup showing the light path through the interferometric cavity comprising a crossed-cylinder lens arrangement. The lower lens is mounted on a spring with a known spring constant.

RESULTS AND DISCUSSION

The measured interaction free energy as a function of the separation between two negatively charged mica surfaces across an aqueous solution of proline at a low concentration (0.07 m) with no added salt is shown in Fig. 2 *A*. Also shown in this figure is a control experiment with pure water. Upon approach, the surfaces repel one another from large separations, with the repulsion increasing in strength as the separation decreases. This repulsion continues to increase down to a surface separation of around 4 nm, at which point a strong attractive force dominates, and the surfaces jump together. The presence of a strong attraction (energy minimum) is confirmed upon separation of the surfaces, where a negative interaction energy of around -1 mJ m^{-2} is required to bring the surfaces out of contact, at which point there is a sudden jump to large surface separations. Compared to the pure water measurement, the repulsive force is of substantially larger magnitude.

To interpret these measurements, we consider the Derjaguin-Landau-Verwey-Overbeek (DLVO) theory of colloidal stability, which states that the total interaction energy between two surfaces is the sum of electrostatic and van der Waals terms. When identical surfaces are interacting across an electrolyte, the repulsive electrostatic term emerges from the overlap of the double layers of countercharge present at each surface and the resulting excess osmotic pressure. This can be captured using the Poisson-Boltzmann equation and weak overlap approximation, combined with suitable boundary conditions. In the present case, we find that neither a constant charge (CC) nor a constant potential boundary condition properly fits the data; rather, it is necessary to consider charge regulation effects arising from adsorption of ions to the surfaces during the approach and concomitant variation of the surface charge and potential with distance. Here, we employ the constant regulation approximation, which has been used to successfully interrogate a range of measurements across aqueous electrolyte solutions (23,24) and allows for linear regulation between the constant potential and CC boundary conditions with a single dimensionless regulation parameter, p, taking values between 0 and 1 (25,26). The value of p provides an indication

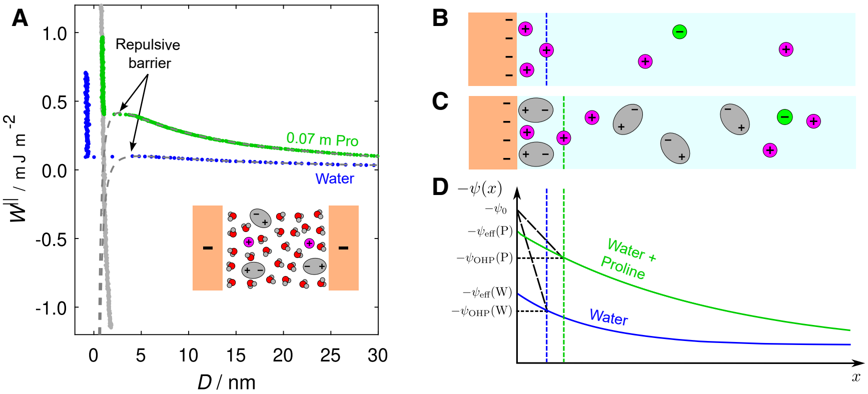

FIGURE 2 (*A*) Measurement of the interaction free energy $W^{\|}$ as a function of surface separation D between negatively charged mica surfaces, made across a solution containing 0.07 m proline (*green*) and pure water (*blue*). For the proline solution, the interaction potential measured upon retraction of the surfaces is also shown in gray. The DLVO fits to both measurements are shown by the gray dashed curves. The same data are presented on a log-linear axes in Fig. S1 (Section S3 of the supporting material). (*B* and *C*) Schematics illustrating the structure of a charged interface in pure water and in the presence of proline zwitterions. (*D*) An illustrative plot of potential ψ as a function of distance from the interface x in both cases. The actual surface potential at the interface ψ_0, the effective potential extrapolated from the electric double-layer potential ψ_{eff}, and the outer Helmholtz plane (OHP) potential ψ_{OHP} are shown; these differ because the decay of potential across the Stern layer is not captured in the Poisson-Boltzmann equation.

of the molecular processes occurring during approach of the surfaces, as will be illustrated later. The attractive van der Waals contribution to the total interaction free energy is calculated using the Hamaker approach, which is discussed further in Section S4 of the supporting material (27). The resulting total interaction energy can be written as shown in Eq. 1:

$$W^{\|} = -\frac{A}{12\pi D^2} + 2\epsilon_0\epsilon_e\kappa_D\psi_{\mathrm{eff}}^2\frac{e^{-\kappa_D D}}{1+(1-2p)e^{-\kappa_D D}}, \quad (1)$$

where A is the Hamaker constant, ϵ_0 is the permittivity of free space, ϵ_e is the electrolyte relative permittivity, ψ_{eff} is the effective surface potential, and κ_D^{-1} is the Debye-Hückel screening length.

Fits to the measurements with pure water and 0.07 m proline in water using Eq. 1 are shown by the gray dashed curves in Fig. 2 *A*. The chosen functional form for the interaction potential provides an accurate fit to the long-range repulsion, as well as providing an attractive force that can explain the jump at small surface separations. The parameters resulting from this and all fits are displayed in Table 1 and reveal a measured Debye-Hückel screening length $\kappa_D^{-1} = 40 \pm 2$ nm.

Despite there being little difference in the Debye screening length between the proline and pure water cases, there is a significant enhancement to the effective surface potential in the presence of proline: $\psi_{\mathrm{eff}} = 62 \pm 5$ mV ($\approx 2.5 k_B T/e$) for 0.07 m proline and $\psi_{\mathrm{eff}} = 38 \pm 14$ mV ($\approx 1.5 k_B T/e$) for pure water. This strong enhancement of the repulsive force with zwitterions in solution is in line with previous studies, where the enhanced ψ_{eff} was attributed to the adsorption of zwitterions at the interface due to the favorable interaction between the negatively charged surface and the strong zwitterion dipole moment (28). The interfacial zwitterions replace the ions that would otherwise be present in the Stern layer, and the cations have a reduced affinity for the plane of negative charge at the edge of the zwitterion layer than for the charged mica interface, thus resulting in a more negative effective potential relative to the pure water measurement. This difference is illustrated schematically in Fig. 2, *B* and *C*, and graphically in Fig. 2 *D*. The enhanced repulsive interaction in the presence of proline relative to pure water is illustrated clearly in Fig. S2 (Section S5 of the supporting material), where both ψ_{eff} and the pre-exponential factor $2\epsilon_0\epsilon_e\kappa_D\psi_{\mathrm{eff}}^2$ are plotted for these measurements.

The fitted charge regulation parameter is found to be $p = 0.90 \pm 0.03$ and indicates that, for 0.07 m proline, the system remains close to the CC boundary during the approach of the surfaces. This is likely a result of the very small concentration of free ions present in the solution (6×10^{-5} M, calculated from κ_D^{-1}) available to adsorb during the approach of the surfaces. In light of this, it is meaningful to report the surface charge σ_{eff}, which can be calculated from the effective surface potential using the Graheme equation, a discussion of which is provided in Section S6 of the supporting material. For this measurement, we calculate the surface charge to be $\sigma_{\mathrm{eff}} = 7.1 \times 10^{-3}$ e nm^{-2}.

Next, we describe experiments at higher proline concentrations. Fig. 3 *A* displays the previously described measurement at a proline concentration of 0.07 m, in addition to measurements performed at elevated concentrations of

TABLE 1 Parameters used for DLVO fits to the measured interaction free energy, $W^{\|}$, for the displayed measurements across the electrolytes containing proline and potassium chloride

Figure	c_{Pro}/m	c_{KCl}/m	ψ_{eff}/mV	κ_D^{-1}/nm	p	$\kappa_{D,\mathrm{pred}}^{-1}$/nm
2	–	–	38 ± 12	65 ± 21	0.88 ± 0.10	–
2 and 3	0.07	–	63 ± 5	40 ± 2	0.90 ± 0.01	–
3	0.35	–	55 ± 2	34 ± 7	0.92 ± 0.02	–
3	0.90	–	49 ± 2	30 ± 5	0.92 ± 0.03	–
4	0.46	0.01	49 ± 3	3.0 ± 0.2	0.61 ± 0.08	3.3
4[a]	–	0.01	29 ± 1	3.8 ± 0.8	1.0 ± 0.0	3.1

For each concentration, the fitted effective surface potential ψ_{eff}, Debye screening length κ_D^{-1}, and charge regulation parameter p are shown. In solutions where KCl is present, the predicted Debye screening length $\kappa_{D,\mathrm{pred}}^{-1}$ is also shown.

[a]The quality of the fit for this measurement is lower than for the others, and as such, the derived parameters may be less reliable.

0.35 and 0.90 m. At all concentrations, we observe a similar long-range repulsion as the surfaces approach. Interestingly, the longer-ranged force between the surfaces across proline solutions is remarkably insensitive to the proline concentration (above a low threshold; in our experiments, the lowest concentration studied is 0.07 m). Higher proline concentrations give rise to more adsorbed layers and stronger short-range forces, but the far field is not affected, as shown clearly in Fig. S2 (supplemental section 5). Therefore, proline could be used to tune the osmotic pressure (Table S3 in Section S7 of the supporting material displays an estimate for all studied solutions) while retaining close regulation of far-field interaction forces.

At the elevated concentrations (0.35 and 0.90 m), however, we observe an additional feature at small surface separations not present at the lowest concentration, which is displayed more clearly in Fig. 3 *B*. In both cases, a hard wall in the interaction profile is observed at an intermediate distance, followed by a simultaneous jump of the surfaces and squeeze out of a molecular layer of thickness ~0.5 nm before the closest approach distance is reached. This step size is consistent with the size of a proline molecule along its axis as obtained from x-ray diffraction structural determination (29). We suggest, therefore, that this structural, non-DLVO feature at small surface separations arises from layers of proline adsorbing with their positively charged ammonium group at the negatively charged mica surface and the negatively charged carboxylate group pointing away from the surface. Our interpretation of proline

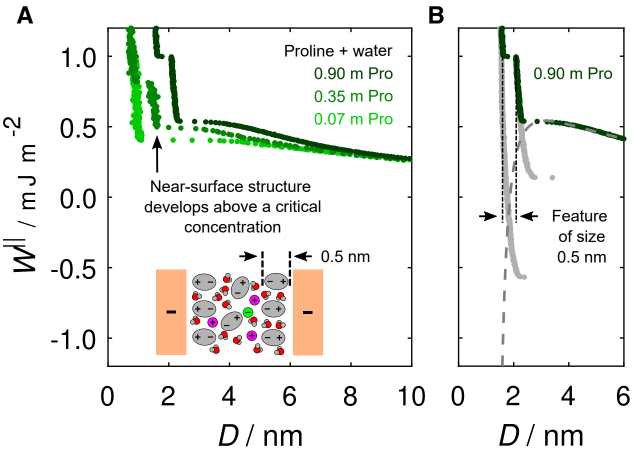

FIGURE 3 (*A*) Measurement of the interaction free energy $W^{||}$ as a function of surface separation D at three different proline concentrations. Additional near-surface features are observed at the higher concentrations (0.35 and 0.90 m), which are not seen at the lower concentration. For clarity, no DLVO fits are shown here, but the associated parameters are displayed in Table 1. (*B*) View of the measurement across the 0.90 m proline solution, concentrated on the near-surface features with the 0.5 nm step indicated. In addition to the approach measurement (*dark green*), the data obtained upon retraction from the two layers are also displayed in gray, which reveal the interaction minimum associated with each layer. The DLVO fit to this measurement is also shown by the gray dashed curve.

forming layers at the interface is consistent with our earlier analysis behind the enhanced effective surface potential, as well as other recent discoveries finding layering of zwitterions at charged interfaces (15).

Next, we present measurements with added salt across a solution containing 0.46 m proline and 0.01 m KCl in Fig. 4; also displayed for comparison is a measurement across a KCl-only solution at the same salt concentration. From the DLVO fit to the long-range repulsion in the proline-KCl mixture, we see an effective surface potential similar to the proline-only cases ($\psi_{\text{eff}} = 49 \pm 5$ mV). In contrast, there is a noticeable difference in the Debye screening length ($\kappa_D^{-1} = 3.0 \pm 0.2$ nm) and the charge regulation parameter ($p = 0.61 \pm 0.08$). The Debye length can be simply attributed to the presence of KCl, matching that predicted for a 1:1 electrolyte solution at the same KCl concentration (Table 1). Clearly, the reduced value of p in the presence of 0.01 m KCl indicates an increased tendency to regulate surface charge during the approach: ions are now more available in solution to adsorb and lower the effective charge. Consistent with this observation, a measurement across 0.33 m proline in the presence of 0.001 m KCl, displayed in Fig. S3 (Section S8 of the supporting material), was found to have an intermediate value between these two cases ($p = 0.84 \pm 0.03$).

Turning to the non-DLVO, structural contribution at small surface separations, we see that, in the presence of salt, the short-range "step" characteristic of squeezing out near-surface molecular layers is now reduced to 0.3 ± 0.1 nm. Such features are reminiscent of those present in measurements across dilute aqueous electrolyte solutions (30). These "hydration forces" were measured above a critical salt concentration—4×10^{-5} M in the case of KCl (31)—intrinsically linking the origin of these forces to the presence of weakly hydrated ions at the mica interface. Despite proline zwitterions being present at much higher concentrations than the KCl ions, the structural feature is now dominated by the molecular packing of hydrated ions at the interface. Indeed, this observation of a shift in the structural feature from proline dominated to water dominated due to the presence of monovalent ions in the solution is consistent with the appearance of charge regulation behavior across the solutions with salt. Our measurements appear to reveal a link between the observed structural features and the charge regulatory behavior: CC behavior prevails in the electric double-layer repulsive regime when proline dominates the near-surface structure, but charge regulation is enhanced when the water-dominated structural features are observed, owing to the presence of adsorbed ions at the interface.

The measurement across the KCl-only solution shown in Fig. 4 displays a similar long-range interaction to the proline-KCl mixture and similarly displays a repulsion due to hydration forces at short range. However, comparing interactions with and without proline, the magnitude of hydration forces is strongly divergent; in the proline-KCl

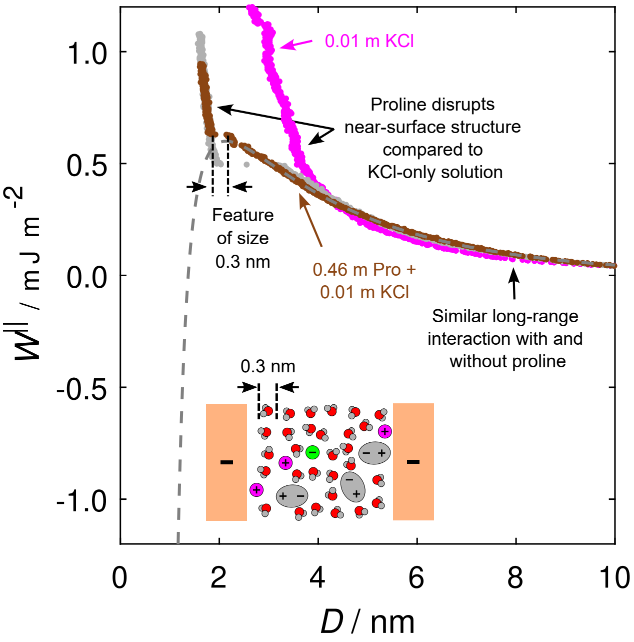

FIGURE 4 Measurement of the interaction free energy $W^{||}$ as a function of surface separation D across a solution containing 0.46 m proline and 0.01 m KCl concentrations (*brown*) and the associated measurement upon retraction of the surfaces (*gray*). The DLVO fit to this measurement is shown by the gray dashed curve. A measurement across a solution containing 0.01 m KCl only (no proline) is displayed in magenta.

mixture, the proline zwitterions act to disrupt the near-surface hydration structure. A similar finding has previously been observed in another zwitterionic osmolyte-salt mixture (28). This observation further illustrates the interplay that exists between the proline and ionic species in determining the nature of the overall interaction.

Finally, we return to examine the values of surface potential and surface charge density apparent in each of our experiments, as displayed in Table S2 (Section S6 of the supporting material). It is noticeable that the values of the determined surface potentials vary only slightly with electrolyte composition in the range 1–3 k_BT. In contrast, the value of the surface charge in the presence of proline is much more variable and sensitive to ion concentration: values range from $\sigma_{eff} \sim 2$ mC m^{-2} with no added KCl to $\sim$4 and $\sim$16 mC m^{-2} in the presence of 0.001 and 0.01 m KCl, respectively. This arises from the intrinsic inverse coupling between σ_{eff} and κ_D^{-1} for (relatively) invariant ψ_{eff}, the latter being set by the ambient thermal energy.

In this work, we have presented a series of experimental measurements across proline-containing aqueous solutions to investigate the role of proline in modulating colloidal interactions. Firstly, our results demonstrate a clear link between proline and an enhanced double-layer repulsion in the absence of salt. Additionally, we find that the short-range interaction, or the non-DLVO structural contribution, is altered by the presence of proline, which forms layers at high concentrations. When salt is present, proline appears to disrupt hydration layers arising from ions at the interface. Finally, with and without salt, the far-field colloidal interaction is stable against varying proline concentration, implying that biomolecular interactions are maintained while osmotic pressure varies over a wide range.

DATA AND CODE AVAILABILITY

All raw data (interaction free energy as a function of distance) are available from the Oxford University Research Archive (https://doi.org/10.5287/ora-00xxnmb7z).

ACKNOWLEDGMENTS

The authors gratefully acknowledge funding from the European Research Council under grant 101001346 ELECTROLYTE. K.J.A. would like to acknowledge support from the Oxford-The Queen's College Graduate Scholarship in partnership with the Clarendon Fund, University of Oxford.

AUTHOR CONTRIBUTIONS

K.J.A., J.E.H., and S.P. designed the research. K.J.A, and J.E.H. performed the research and analyzed the data. K.J.A., J.E.H., and S.P. wrote the manuscript.

DECLARATION OF INTERESTS

The authors declare no competing interests.

SUPPORTING MATERIAL

Supporting material can be found online at https://doi.org/10.1016/j.bpj.2025.09.043.

REFERENCES

1. Yancey, P. H., M. E. Clark, …, G. N. Somero. 1982. Living with Water Stress: Evolution of Osmolyte Systems. *Science.* 217:1214–1222. https://www.science.org.
2. Yoshiba, Y., T. Kiyosue, …, K. Shinozaki. 1997. Regulation of Levels of Proline as an Osmolyte in Plants under Water Stress. *Plant Cell Physiol.* 38:1095–1102. https://academic.oup.com/pcp/article/38/10/1095/1847190.
3. Slama, I., C. Abdelly, …, A. Savouré. 2015. Diversity, distribution and roles of osmoprotective compounds accumulated in halophytes under abiotic stress. *Ann. Bot.* 115:433–447. https://doi.org/10.1093/aob/mcu239.
4. Szabados, L., and A. Savouré. 2010. Proline: a multifunctional amino acid. *Trends Plant Sci.* 15:89–97.
5. Hayat, S., Q. Hayat, …, A. Ahmad. 2012. Role of proline under changing environments. *Plant Signal. Behav.* 7:1456–1466. https://www.tandfonline.com/doi/abs/10.4161/psb.21949.
6. Shafi, A., I. Zahoor, and U. Mushtaq. 2019. Proline accumulation and oxidative stress: Diverse roles and mechanism of tolerance and adaptation under salinity stress. *Salt Stress, Microbes, and Plant Interactions: Mechanisms and Molecular Approaches.* 2:269–300. https://link.springer.com/chapter/10.1007/978-981-13-8805-7_13.
7. Grgac, R., J. Rozsypal, …, V. Koštál. 2022. Stabilization of insect cell membranes and soluble enzymes by accumulated cryoprotectants during freezing stress. *Proc. Natl. Acad. Sci. USA.* 119:e2211744119. https://www.pnas.org/doi/abs/10.1073/pnas.2211744119.

8. Stewart, G. R., and J. A. Lee. 1974. The role of proline accumulation in halophytes. *Planta*. 120:279–289. https://link.springer.com/article/10.1007/BF00390296.

9. Wennerström, H., and M. Oliveberg. 2022. On the osmotic pressure of cells. *QRB Discovery*. 3:e12. https://www.cambridge.org/core/journals/qrb-discovery/article/on-the-osmotic-pressure-of-cells/72AA0FAD0A150BE55EA5519490C126B7.

10. Deole, R., and W. D. Hoff. 2020. A potassium chloride to glycine betaine osmoprotectant switch in the extreme halophile Halorhodospira halophila. *Sci. Rep*. 10:3383. https://www.nature.com/articles/s41598-020-59231-9.

11. Liang, X., L. Zhang, …, D. F. Becker. 2013. Proline mechanisms of stress survival. *Antioxidants Redox Signal*. 19:998–1011. https://www.liebertpub.com/doi/10.1089/ars.2012.5074.

12. El Moukhtari, A., C. Cabassa-Hourton, …, A. Savouré. 2020. How Does Proline Treatment Promote Salt Stress Tolerance During Crop Plant Development? *Front. Plant Sci*. 11:1127.

13. Govrin, R., S. Tcherner, …, U. Sivan. 2018. Zwitterionic Osmolytes Resurrect Electrostatic Interactions Screened by Salt. *J. Am. Chem. Soc*. 140:14206–14210. https://pubs.acs.org/doi/full/10.1021/jacs.8b07771.

14. Govrin, R., T. Obstbaum, and U. Sivan. 2019. Common Source of Cryoprotection and Osmoprotection by Osmolytes. *J. Am. Chem. Soc*. 141:13311–13314. https://doi.org/10.1021/jacs.9b06727.

15. Ridwan, M. G., B. R. Shrestha, …, H. Mishra. 2022. Zwitterions Layer at but Do Not Screen Electrified Interfaces. *J. Phys. Chem. B*. 126:1852–1860. https://pubs.acs.org/doi/full/10.1021/acs.jpcb.1c10388.

16. Winkler, P. M., C. Siri, …, F. Stellacci. 2024. Modulating Weak Protein-Protein Cross-Interactions by the Addition of Free Amino Acids at Millimolar Concentrations. *J. Phys. Chem. B*. 128:7199–7207. https://pubs.acs.org/doi/full/10.1021/acs.jpcb.4c01086.

17. Xu, X., and F. Stellacci. 2024. Amino Acids and Their Biological Derivatives Modulate Protein-Protein Interactions in an Additive Way. *J. Phys. Chem. Lett*. 15:7154–7160. https://pubs.acs.org/doi/full/10.1021/acs.jpclett.4c01175.

18. Xu, X., A. A. Rebane, …, F. Stellacci. 2024. Amino acids modulate liquid-liquid phase separation in vitro and in vivo by regulating protein-protein interactions. *Proc. Natl. Acad. Sci. USA*. 121:e2407633121. https://www.pnas.org/doi/abs/10.1073/pnas.2407633121.

19. Hayler, H. J., T. S. Groves, …, S. Perkin. 2024. The surface force balance: direct measurement of interactions in fluids and soft matter. *Rep. Prog. Phys*. 87:046601. https://singdirect.iopscience.iop.org/article/10.1088/1361-6633/ad2b9bhttps://singdirect.iopscience.iop.org/article/10.1088/1361-6633/ad2b9b/meta.

20. Derjaguin, B. 1934. Untersuchungen über die Reibung und Adhäsion, IV - Theorie des Anhaftens kleiner Teilchen. *Kolloid Z*. 69:155–164. https://link.springer.com/article/10.1007/BF01433225.

21. Haynes, W. M. 2014. CRC Handbook of Chemistry and Physics, 95th edition. CRC Press, pp. 7–1.

22. Romano, E., F. Suvire, …, M. A. A. Molina. 2006. Dielectric properties of proline: Hydration effect. *J. Mol. Liq*. 126:43–47. https://www.sciencedirect.com/science/article/pii/S0167732205000759?via.

23. Trefalt, G., S. H. Behrens, and M. Borkovec. 2016. Charge Regulation in the Electrical Double Layer: Ion Adsorption and Surface Interactions. *Langmuir*. 32:380–400. https://pubs.acs.org/doi/full/10.1021/acs.langmuir.5b03611.

24. Smith, A. M., M. Borkovec, and G. Trefalt. 2020. Forces between solid surfaces in aqueous electrolyte solutions. *Adv. Colloid Interface Sci*. 275:102078.

25. Carnie, S. L., and D. Y. C. Chan. 1993. Interaction Free Energy between Plates with Charge Regulation: A Linearized Model. *J. Colloid Interface Sci*. 161:260–264.

26. Pericet-Camara, R., G. Papastavrou, …, M. Borkovec. 2004. Interaction between charged surfaces on the poisson - Boltzmann level: The constant regulation approximation. *J. Phys. Chem. B*. 108:19467–19475. https://pubs.acs.org/doi/full/10.1021/jp0473063.

27. Israelachvili, J. N. 2011. Intermolecular and Surface Forces, third edition. Academic Press.

28. Hallett, J. E., K. J. Agg, and S. Perkin. 2023. Zwitterions fine-tune interactions in electrolyte solutions. *Proc. Natl. Acad. Sci. USA*. 120:e2215585120. https://www.pnas.org/doi/abs/10.1073/pnas.2215585120.

29. Koenig, J. J., J. M. Neudörfl, …, M. Breugst. 2018. Redetermination of the solvent-free crystal structure of l -proline. *Acta Crystallogr. E Crystallogr. Commun*. 74:1067–1070. https://scripts.iucr.org/cgi-bin/paper?eb2008.

30. Pashley, R. M., and J. N. Israelachvili. 1984. Molecular layering of water in thin films between mica surfaces and its relation to hydration forces. *J. Colloid Interface Sci*. 101:511–523.

31. Pashley, R. M. 1981. DLVO and hydration forces between mica surfaces in Li+, Na+, K+, and Cs+ electrolyte solutions: A correlation of double-layer and hydration forces with surface cation exchange properties. *J. Colloid Interface Sci*. 83:531–546.

Biophysical Journal, Volume *124*

Supplemental information

The influence of proline on surface interactions in aqueous solutions

Kieran J. Agg, James E. Hallett, and Susan Perkin

1 Additional experimental details

In our measurements, the surfaces are initially prepared by manually cleaving mica (optical-grade ruby muscovite mica, S & J Trading Inc.) in a dust free environment, yielding atomically smooth facets of area on the magnitude of several cm^2 and thickness of 2 to 4 $\mathrm{\mu m}$. These are subsequently adhered on a freshly cleaved mica substrate and back-silvered via thermal evaporation to a layer thickness of $\sim$ 45 nm (99.9999%, Alfa Aesar) using an Auto 306 thermal evaporator (HHV Ltd.). The mica substrates are then stored under vacuum until use. Two of these mica pieces are glued using an epoxy resin (EPON™ Resin 1004F, Miller-Stephenson Chemical Co., Inc.), silver-side down, to cylindrical glass lenses of radius $R \approx 10$ mm. The lenses are mounted in a crossed-cylinder orientation, with the lower lens located on a leaf spring of pre-calibrated spring constant. A white light source is incident on an interferometric cavity which is formed by the two silver mirrors, and the light emergent from this cavity is passed into a grating spectrometer to measure the resulting interference pattern which appears as fringes of equal chromatic order (FECO). Analysis of the FECO can determine the surface separation D and the radius of the surfaces R. The forces exerted between the two surfaces can be calculated by measuring the deflection of the lower lens on the leaf spring. A calibration measurement of the mica thickness is performed by bringing the surfaces together in air, which is necessary for the determination of absolute surface separation.

The electrolyte solution of interest is then injected between the two lenses to form a bridging droplet ($\sim$ 0.3 mL). Equilibrium forces were measured by driving the surfaces toward each other at a constant velocity (ranging between 2 and 10 nm s^{-1}) using a mechanical or piezoelectric drive. A measurement performed whilst approaching the surfaces yields data for the monotonic repulsive force at larger surface separations and structural forces at smaller surface separations, and retracting the surfaces can reveal the nature of one or more potential minima.

2 Experimental error analysis

In this work, we choose to present individual surface force measurements for different sample compositions, as these most clearly show features of interest at small length scales. The random error in the measurement of an individual data point within a single run is very small - approximately $\sim$ 0.1 nm in D and $\sim$ 0.01 mJ m^{-2} in $W^{||}$ - thus allowing these molecular-scale features to be visible. These errors arise from the uncertainty in the tracking of the fringes of equal chromatic order (FECO) through which distance is calculated, and in the value of the spring constant from which forces or interaction energies are determined.

If multiple measurement runs were averaged, these subtle features would be smeared out. Systematic errors are present across runs, of magnitude $\sim$ 0.5 nm in D and $\sim$ 0.1 mJ m^{-2}, which arise from errors in optical alignment, measurement of the radius of curvature, temperature and concentration of samples. For the measurements presented in this work for a given composition, no significant differences appear between individual measurement runs, with no dependence on equilibration time (between adjacent runs), time after introducing the liquid between the mica surfaces or the microscopic contact spot between the two crossed cylinders. Between runs, there are small variations in the forces at which jumps due to a spring instability occur and in the magnitude of long range interactions, but changes in distance over which features occur (e.g. molecular layer thicknesses) and screening lengths are more reproducible.

3 Interaction energies presented on log-linear axes

Figs. 2 (A), 3 (A) and 4 from the main text are reproduced in Fig. S1 on log-linear axes. Presenting the data in this manner aids the visualisation of the range and magnitude of the interaction.

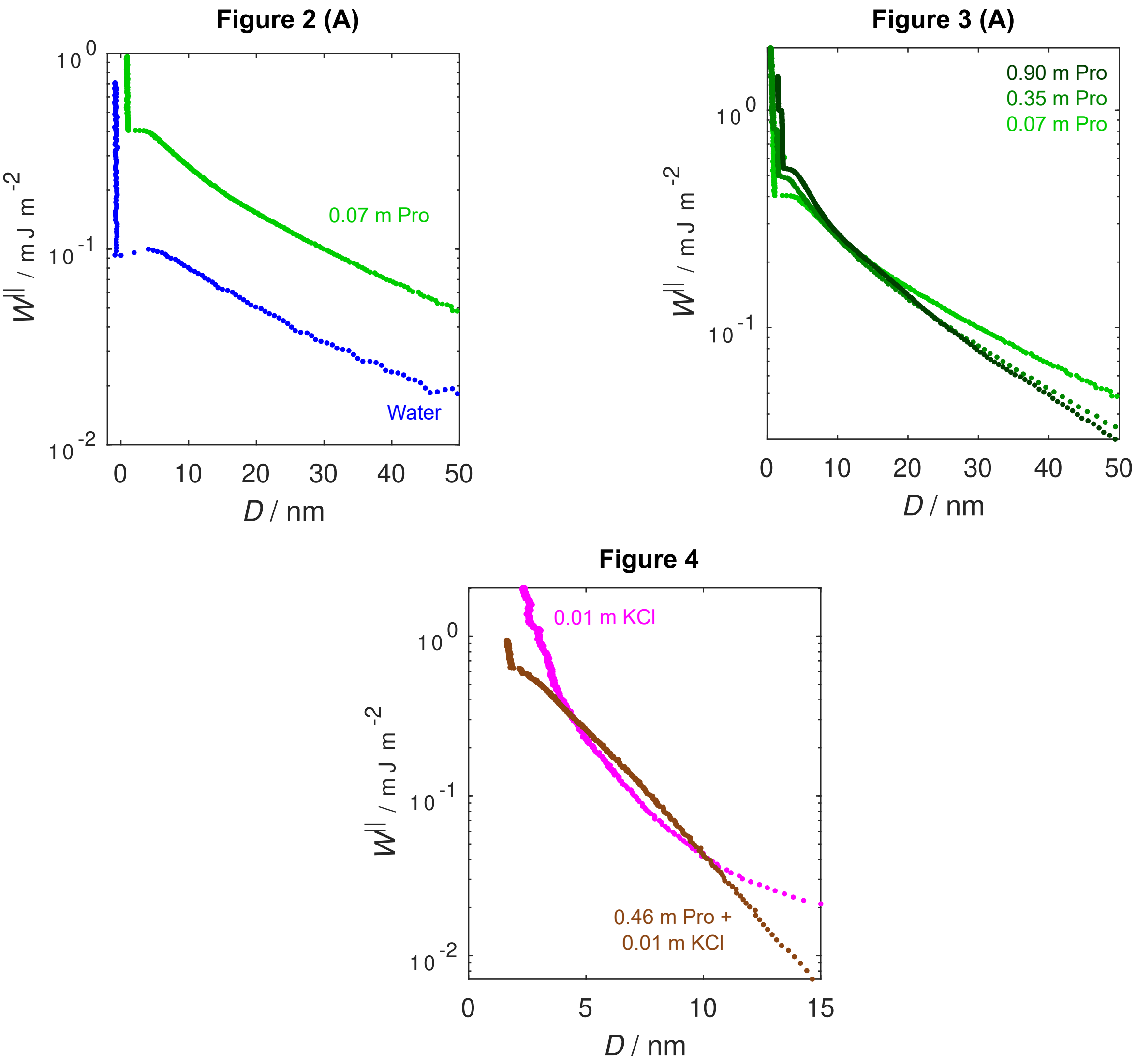

Figure S1. Data from Fig. 2 (A), Fig. 3 (A) and Fig. 4 in the main text are reproduced here on log-linear axes.

4 Calculation of Hamaker constants

For the DLVO fits in this work, we calculate the Hamaker constants between mica surfaces across electrolytes of given composition using the Lifshitz theory with the approach detailed by Israelachvili, as shown in Eq. S1 [1]:

$$A = \frac{3}{4}kT\left(\frac{\epsilon_{\mathrm{m}}-\epsilon_{\mathrm{e}}}{\epsilon_{\mathrm{m}}+\epsilon_{\mathrm{e}}}\right)^2 + \frac{3h\nu_{\mathrm{e}}}{16\sqrt{2}}\frac{(n_{\mathrm{m}}^2-n_{\mathrm{e}}^2)^2}{(n_{\mathrm{m}}^2+n_{\mathrm{e}}^2)^{\frac{3}{2}}} \tag{S1}$$

where k is the Boltzmann constant; T is the temperature (295 K); ϵ_{m} and ϵ_{e} are the relative permittivities of mica and electrolyte, respectively; h is the Planck constant; ν_{e} is the plasma frequency of the free electron gas; and n_{m} and n_{e} are the refractive indices of mica and electrolyte, respectively [1,2]. The relative permittivity of mica was taken as 2, the frequency of the free electron gas was taken as 3×10^{15} s^{-1}, and the refractive index of mica was taken as 1.6 [1]. An Abbe 60 Refractometer (Bellingham and Stanley) and a sodium lamp of wavelength 589.3 nm was used to measure the refractive indices of the measured solutions.

The relative permittivity of the electrolyte was estimated using Eq. S2:

$$\epsilon_{\mathrm{e}} = \epsilon_{\mathrm{w}} + \delta_{\mathrm{Pro}}c_{\mathrm{Pro}} + \delta_{\mathrm{KCl}}c_{\mathrm{KCl}} \tag{S2}$$

where ϵ_{w} is the relative permittivity of water, δ is the dielectric increment or decrement and c is the solute concentration. Here, the dielectric increments or decrements were obtained from literature, and we have taken δ_{Pro} to be 21.0 / m [3], and δ_{KCl} to be -8.85 / m. [4]

The calculated Hamaker constants and associated variables are displayed in Table S1.

Table S1. Parameters used for Hamaker constant calculations. Concentrations of proline c_{Pro} and KCl c_{KCl}, electrolyte refractive index n_{e}, electrolyte relative permittivity ϵ_{e} and Hamaker constant A are shown.

Figure	c_{Pro} / m	c_{KCl} / m	n_{e}	ϵ_{e}	A / 10^{-20} J
2	-	-	1.3300	78.4	2.11
2 & 3	0.07	-	1.3339	79.9	2.05
3	0.35	-	1.3394	85.5	1.98
3	0.90	-	1.3507	97.3	1.84
S3	0.33	0.001	1.3382	85.3	2.00
4	0.46	0.010	1.3414	88.0	1.96
S3	-	0.001	1.3320	78.4	2.08
4	-	0.010	1.3321	78.3	2.08

5 Surface potential as a function of proline concentration

For the proline-only aqueous solutions discussed in this work (Figs. 2 (A) and 3 (A)), the effective surface potential ψ_{eff} obtained from the DLVO fits, as tabulated in Table 1, is plotted as a function of proline concentration in Fig. S2 (A). Since the magnitude of the repulsive barrier present in the interaction profile is not solely dependent on ψ_{eff}, the pre-exponential factor $2\epsilon_0\epsilon_{\mathrm{e}}\kappa_{\mathrm{D}}\psi_{\mathrm{eff}}^2$ is plotted in Fig. S2 (B). This figure clearly demonstrates the enhanced repulsive barrier in the presence of proline relative to pure water, but that the magnitude is not significantly changed by dialling up the proline concentration.

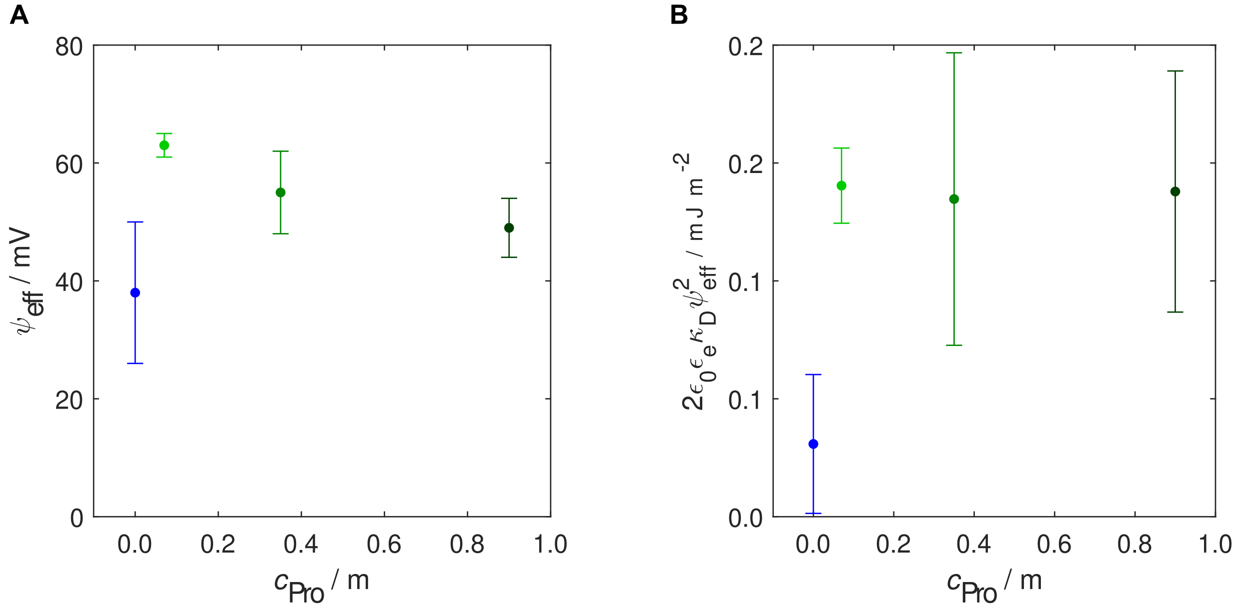

Figure S2. (A) Effective surface potential ψ_{eff} as a function of proline concentration. (B) The pre-exponential factor $2\epsilon_0\epsilon_{\mathrm{e}}\kappa_{\mathrm{D}}\psi_{\mathrm{eff}}^2$ as a function of proline concentration.

6 Calculations of surface charge density

In order to interconvert between the fitted value for effective surface potential ψ_{eff} and surface charge σ, we use the following relations discussed in the review article by Smith *et al.*[5]

For weakly charged surfaces, the effective surface charge density σ_{eff} can be calculated using the Debye-Hückel relation shown in Eq. S3.

$$\sigma_{\mathrm{eff}} = \epsilon_0 \epsilon \kappa_{\mathrm{eff}} \psi_{\mathrm{eff}} \tag{S3}$$

For more highly charged surfaces, the Poisson-Boltzmann equation must be invoked. Eq. S4 relate the effective surface potential ψ_{eff}, extracted from our measurements, and diffuse layer potential ψ_{dl}, the potential at the origin of the diffuse layer, across a symmetric 1:1 electrolyte.

$$\psi_{\mathrm{eff}} = \frac{4kT}{e} \tanh\left(\frac{e\psi_{\mathrm{dl}}}{4kT}\right) \tag{S4}$$

A surface charge density σ can be calculated from the diffuse layer potential ψ_{dl} using the Graheme equation, shown in Eq. S5 for a symmetric 1:1 electrolyte.

$$\sigma = \frac{2kT\epsilon_0\epsilon\kappa_{\mathrm{D}}}{e} \sinh\left(\frac{e\psi_{\mathrm{dl}}}{2kT}\right) \tag{S5}$$

The surface charge densities and surface potentials calculated from the fitted effective surface potential ψ_{eff} and Eqs. S3-S5 are displayed in Table S2.

Table S2. Table of calculated constants for surface potentials and charge densities. Concentrations of proline c_{Pro} and KCl c_{KCl}, fitted effective surface potential ψ_{eff}, effective surface charge density σ_{eff}, double layer potential ψ_{dl} and surface charge density σ are shown. †The quality of the fit for this measurement is lower than for the others, and as such, the derived parameters may be less reliable.

Figure	c_{Pro}/ m	c_{KCl}/ m	κ_{D}^{-1} / nm	ψ_{eff} / mV	σ_{eff} / 10^{-3} e nm^{-2}	ψ_{dl} / mV	σ / 10^{-3} e nm^{-2}
2	-	-	65	38	2.5	40	2.9
2 & 3	0.07	-	40	63	7.1	74	11
3	0.35	-	34	55	7.6	61	11
3	0.90	-	30	49	8.8	54	11
S3	0.33	0.001	11	49	21	53	27
4	0.46	0.010	3.0	49	78	53	100
S3	-	0.001	9.5	56	26	63	36
4 †	-	0.010	3.8	29	33	30	36

7 Osmotic pressures of studied solutions

The osmotic pressure Π can be calculated for an ideal solution using the van't Hoff equation, shown in Eq. S6:

$$\Pi = \sum icRT \quad \text{(S6)}$$

which is summed over all solutes, and where i is the van't Hoff factor, c is the solute concentration, R is the ideal gas constant and T is the temperature. The van't Hoff factor i was taken as 1 for proline and 2 for KCl, and the solute concentration in molar was approximated to be the measured value in molal, valid for small concentrations. The osmotic pressure of all solutions studied in this work is shown in Table S3.

Table S3. Osmotic pressures of all solutions studied in this work. Concentrations of proline c_{Pro} and KCl c_{KCl} and osmotic pressure Π are shown.

Figure	c_{Pro} / m	c_{KCl} / m	Π / atm
2	-	-	-
2 & 3	0.07	-	1.7
3	0.35	-	8.6
3	0.90	-	22.0
S3	0.33	0.001	8.1
4	0.46	0.010	11.7
S3	-	0.001	0.05
4	-	0.010	0.49

8 Surface forces measurements at additional concentrations

A measurement across a solution containing 0.33 m proline and 0.001 m KCl is displayed in Fig. S3. The associated DLVO and Hamaker constant parameters are displayed in Table S4 and S1, respectively, along with those from the main article. This measurement appears qualitatively similar to the high concentration proline measurements with a long-range repulsion, van der Waals attractive jump and structural feature in the approach interaction profile. However, the structural jump is of size 0.3 nm, and thus likely dominated by the packing of water molecules at the interface, akin to the structural feature observed in the measurement at the 0.01 m KCl concentration (Fig. 4). The effective surface potential of the long-range attraction is similarly enhanced relative to the pure water case ($\psi_{\text{eff}} = 49 \pm 2$ mV); the Debye screening length is consistent with that for a 1:1 electrolyte containing 0.001 m KCl ($\kappa_{\text{D}}^{-1} = 11 \pm 1$ nm); and the charge regulation parameter is an intermediate value between the proline-only measurements and the 0.01 m KCl measurement, as discussed in the main text ($p = 0.84 \pm 0.03$) .

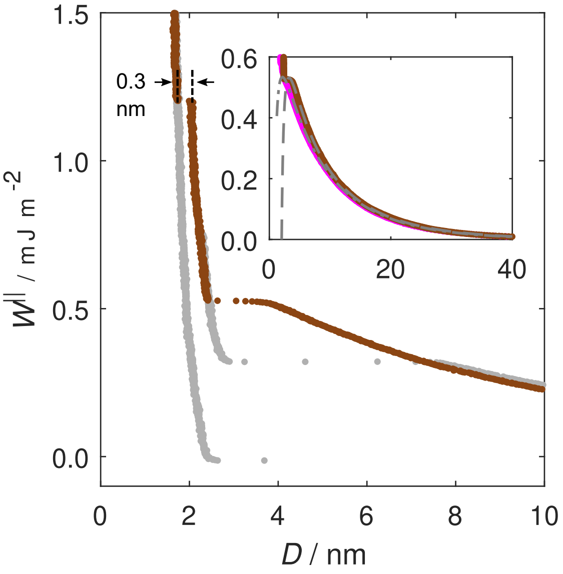

Figure S3. Measurement of the interaction potential ($W^{||}$) across proline solutions as a function of surface separation (D) between negatively charged mica surfaces, made across an electrolyte containing 0.33 m proline and 0.001 m KCl. The measurement of interaction potential measured upon approach of the surfaces is shown in brown, and the retractions from the two layers are shown in grey. In the inset, this proline-KCl measurement (brown) is compared to one across a pure 0.001 m KCl solution (magenta) in addition to the associated DLVO fits (grey).

d

Table S4. Parameters used for DLVO fits to the measured interaction free energy $W^{||}$, for the displayed measurements across the electrolytes containing proline and potassium chloride. For each concentration, the fitted effective surface potential ψ_{eff}, Debye screening length κ_{D}^{-1} and charge regulation parameter p are shown. In solutions where KCl is present, the predicted Debye screening length $\kappa_{\text{D, pred}}^{-1}$ is also shown.

c_{Pro}/ m	c_{KCl}/ m	ψ_{eff} / mV	κ_{D}^{-1} / nm	p	D_0 / nm	$\kappa_{\text{D, pred}}^{-1}$ / nm
0.33	0.001	49 $\pm$ 2	11 $\pm$ 1	0.84 $\pm$ 0.03	1.4 $\pm$ 0.1	10
-	0.001	56 $\pm$ 4	9.5 $\pm$ 0.9	0.72 $\pm$ 0.06	0.2 $\pm$ 0.3	9.7

Supporting References

[1] J. N. Israelachvili, Intermolecular and Surface Forces, Academic Press, 3rd edn, 2011.

[2] E. M. Lifshitz, Journal of Experimental and Theoretical Physics, 1956, **2**, 73–83.

[3] T. Chen, G. Hefter and R. Buchner, Journal of Physical Chemistry A, 2003, **107**, 4025–4031.

[4] J. Kirchnerova, P. G. Parrel, J. T. Edward, T. Shida, W. H. Hamill, J. C. Phys, J. L. Franklin, J. G. Dillard, M. Rosenstock, J. T. Herron, K. Draxl, F. H. Field and P. G. Farrell, Journal of Physical Chemistry, 1976, **80**, 1974–1980.

[5] A. M. Smith, M. Borkovec and G. Trefalt, Advances in Colloid and Interface Science, 2020, **275**, 102078.
